# The phenomenon of “processual transformation” as an indicator of the vulnerability of patients of late age in forensic psychiatry

**DOI:** 10.1192/j.eurpsy.2023.1869

**Published:** 2023-07-19

**Authors:** P. Liubov

**Affiliations:** Department of gerontopsychiatry, Moscow Research Institute of Psychiatry - branch of the V. Serbsky National Medical Research Center for Psychiatry and Narcology, Moscow, Russia, Moscow, Russian Federation

## Abstract

**Introduction:**

Demographic ageing in the world population is accompanied by increased negative social trends towards elderly persons, age discrimination, cruel treatment and violence.

There is an increase in crimes against the elderly related to fraud with their property, physical violence by children because of claims on the property of their parents.

We observe a growing number of property transactions committed under the influence of fraud, deceit, difficult life circumstances. There is an increasing number of elderly, deprived of legal capacity.

**Objectives:**

In order to identify the biological, socio-psychological, legal and victimological determinants we examined 235 patients of late age who underwent forensic psychiatric examination in a criminal and civil process.

**Methods:**

Revealed: «non-dementia» mental disorders - in 45.5%, psychosis - in 7.7%, dementia - in 46.8%.

**Results:**

Patients can simultaneously and consistently participate in criminal and civil process in the status of accused, victims, plaintiffs, defendants, people in respect of whom the incapacity is determined.

The reasons for initiating these cases were often interrelated, the result of a long-term family conflict, often related to the disposal of property.

**Conclusions:**

Such a change in the processual status of patients was characteristic of the late age, reflected their victimization.

This phenomenon was called “processual transformation”.

The probability of " processual transformation " imposes special requirements on the quality of examination of patients of later age. They should be as objective and reliable.

Since the previous expert opinions may affect the subsequent expert opinions in other processes.This phenomenon are legal determinants that can have a disadaptive effect on the late age.

**Disclosure of Interest:**

None Declared

